# Bright’s Disease

**Published:** 1884-04

**Authors:** 


### BRIGHT'S DISEASE.

ON THIS Subject, Dr. Alex. De Borra, of Crystal Springs, N. Y., writes to the *Scientific American*, that, after years of practical test of the milk diet for Bright's disease, he has a long list of cases in which he has made perfect cures. Great care is taken to get absolutely pure skimmed milk from healthy and well-fed cows, and no other food of any kind is given after the patient can bear five pints of milk a day. Up to this point, and until the stomach is able to take care of so much, is found to be the most trying period in this treatment; but no other medicine is given, and hand and hair-glove rubbing is daily administered.

A correspondent takes exception to the claim made, that no drug of any therapeutic value in that disease has yet been discovered, and gives a recipe which, he claims, has affected cures in Bright's disease as well as in dropsy, during fifteen years: "Take a double handful of the dry pods of the common white soup bean or corn bean, to three quarts of water; boil slowly for three hours, until it is reduced to three pints. Take hot or cold. Use no other drink

